# Clinical Implication of Prone Position Electrocardiograms in Patients With COVID‐19

**DOI:** 10.1002/clc.70082

**Published:** 2025-01-18

**Authors:** Naoya Kataoka, Teruhiko Imamura

**Affiliations:** ^1^ Second Department of Internal Medicine University of Toyama Toyama Japan

**Keywords:** Asian, electrophysiology, pneumonia

To Editor,

The posterior lead can be estimated in the supine position using a specific electrocardiogram (ECG) algorithm. Makarawate and colleagues directly measured the posterior lead ECG in the prone position [1]. They demonstrated that a prolonged QTc interval in the prone position correlated with higher APACHE II scores in patients with COVID‐19. Several concerns have been raised regarding their findings.

The QTc interval is strongly correlated with heart rate, as heart rate is a factor in the formula used to calculate the QTc interval. With a fixed QT interval, the QTc interval increases with an elevated heart rate. The authors observed an increase in heart rate in the prone position compared to the standard position [1]. Caution must be exercised in interpreting QTc interval in the prone position, because most of the evidence is constructed from QTc interval in the standard position.

The authors evaluated the impact of ECG findings obtained in the prone position in patients with COVID‐19 [1]. However, some of these patients had acute coronary syndrome, which likely influenced the ECG patterns [2]. It would be more ideal to exclude such patients from the analysis to focus on the primary concern. Additionally, patients with atrial fibrillation were included. The QTc interval is generally overestimated during atrial fibrillation when calculated using the Bazett formula [3].

The authors identified a QTc interval cutoff of 460 ms to predict an APACHE II score > 12. A QTc interval exceeding 460 ms is typically indicative of long QT syndrome, which is commonly observed in patients with electrical disorders or myocardial injury. These individuals likely exhibit severe systemic conditions, explaining why they were treated in the prone position. The clinical utility of ECG measurements in the prone position remains unclear.

## Disclosure

The authors have nothing to report.

## Data Availability

The authors have nothing to report.

## References

[1] P. Makarawate , K. Chimtim , T. Mitsungnern , et al., “Comparison of Standard and Prone‐Position Electrocardiograms in COVID‐19 Patients With Pulmonary Complications: Correlations and Implications,” Clinical Cardiology 47 (2024). 10.1002/clc.70024 PMC11440021 39344374

[2] D. N. Kenigsberg , S. Khanal , M. Kowalski , and S. C. Krishnan , “Prolongation of the QTc Interval is Seen Uniformly During Early Transmural Ischemia,” Journal of the American College of Cardiology 49 (2007): 1299–1305. 17394962 10.1016/j.jacc.2006.11.035

[3] Y. Yu , S. Wen , Y. Ruan , et al., “Impact of Heart Rate and Rhythm on Corrected QT Interval During Paroxysmal Atrial Fibrillation,” American Journal of Cardiology 168 (2022): 64–70. 35065798 10.1016/j.amjcard.2021.12.016

